# Asymmetric Divergence in Transmitted SNPs of DNA Replication/Transcription and Their Impact on Gene Expression in Polyploid *Brassica napus*

**DOI:** 10.3389/fgene.2021.756172

**Published:** 2021-11-11

**Authors:** Minqiang Tang, Juanling Li, Xu Hu, Lu Sun, MMU Helal, Jianguo Chen, Yuanyuan Zhang

**Affiliations:** ^1^ Key Laboratory of Germplasm Innovation of Tropical Special Forest Trees and Ornamental Plants (Ministry of Education), College of Forestry, Hainan University, Haikou, China; ^2^ The Key Laboratory of Biology and Genetic Improvement of Oil Crops, The Ministry of Agriculture, Oil Crops Research Institute of Chinese, Academy of Agricultural Sciences, Wuhan, China; ^3^ School of Life Sciences, Hubei University, Wuhan, China

**Keywords:** polyploidization, gene expression dominance, asymmetrical evolution, *Brassica napus*, transmitted variation

## Abstract

The marked increase in plant genomic data has provided valuable resources for investigating the dynamic evolution of duplicate genes in polyploidy. *Brassica napus* is an ideal model species for investigating polyploid genome evolution. The present study comprehensively analyzed DNA and RNA variation of two representative *B. napus* inbredlines, Zhongshuang11 and Zhongyou821, and we investigated gene expression levels of A_n_ and C_n_ subgenomes in multiple tissues of the two lines. The distribution of transmitted single nucleotide polymorphisms (SNPs) was significantly different in two subgenomes of *B. napus*. Gene expression levels were significantly negatively correlated with number of variations in replication and transcription of the corresponding genes, but were positively correlated with the ratios of transmitted SNPs from DNA to RNA. We found a higher density of SNP variation in A_n_ than that in C_n_ during DNA replication and more SNPs were transmitted to RNA during transcription, which may contribute to A_n_ expression dominance. These activities resulted in asymmetrical gene expression in polyploid *B. napus.* The SNPs transmitted from DNA to RNA could be an important complement feature in comparative genomics, and they may play important roles in asymmetrical genome evolution in polyploidy.

## Introduction

Polyploidy is an important part of the history of natural plant species, and has promoted the domestication of cultivated crop species (Chen et al., 2018). Genome sequencing and comparative genomic analysis have identified ∼50 polyploidization events across plant phylogenetic trees to date (Vanneste et al., 2014; Van de Peer et al., 2017; Chen et al., 2018). Polyploidization is considered the primary driver of plant species diversification and plays an important role in plant genome evolution. Polyploidy has become a popular research topic in plant science due to the increased number of polyploidization events identified in plants. Subgenome dominance, defined as gene fractionation bias and expression dominance between homoeologous genes from different subgenomes, is ubiquitous in polyploidy, especially in allopolyploids. The features of transposable elements (TEs) (Freeling et al., 2012; Bottaniet al., 2018), DNA methylation (Hollister and Gaut 2009), gene density (Cheng et al., 2012), alternative splicing forms of genes (Liu et al., 2014), and single nucleotide polymorphism (SNP)-marked phenotypic diversity (Renny-Byfield et al., 2017) are substantially associated with subgenome dominance. Although understanding the mechanism of subgenome dominance has progressed, further investigation is needed to fully understand the subgenome dominance and polyploidy evolution.

Asymmetric evolution has been confirmed in many plant species since it was originally identified in the mesopolyploid *Brassica oleracea* (Liu et al., 2014). Genes in the dominant subgenome (maize 1) of maize accounted for more trait variations than those in maize 2, indicating asymmetric contributions of different subgenomes (Renny-Byfield et al., 2017). A comparison of the distribution of positively selected genes and fiber-related quantitative trait loci revealed that the A_t_ subgenome was selected for fiber improvement genes, and the D_t_ subgenome was selected for stress tolerance genes (Zhang et al., 2015b). Asymmetrical changes in wheat gene expression, small RNAs, and chromatin in subgenomes have been identified in resynthesized wheat allotetraploids (Jiao et al., 2018)*. B. napus,* a recent allotetraploid species derived from *B. rapa* (AA, 2n = 20) (Wang et al., 2011) and *B. oleracea* (CC, 2n = 18) (Liu et al., 2014), is ideal for studying the effects of SNPs in asymmetric subgenomes. Investigations into asymmetric subgenomes have progressed considerably, and the gaining knowledge has played an important role in guiding genetic improvements in *B. napus*. C_n_ has large chromosome regions associated with important seed quality traits of rapeseed and less diversity than A_n_ (Qian et al., 2014; Tang et al., 2019). A_n_ contains a higher density of SNPs and insertion/deletions (InDels) than C_n_ of *B. napus*, and is consistent with a faster linkage disequilibrium (LD) decay rate of A_n_ than that of C_n_ (Wu et al., 2019). Expression levels of the genes in the A_n_ were significantly higher than those in the C_n_ subgenome, besides, A_n_ possesses a higher level of active epigenetic marks and lower level of repressive epigenetic marks (Zhang et al., 2021). A_n_ had a more homoeolog expression bias and expression level dominance than those of C_n_ in resynthesized *B. napus* (Wu et al., 2018). Currently, asymmetric subgenomes and asymmetric evolution have become a hotspot in polyploid research and a focus of plant science research. However, the effects of SNPs transmitted from DNA to RNA during asymmetric evolution have not been reported.

Gene expression is the process through which information from a gene is used to synthesize a functional gene that enables the generation of end products, proteins, or non-coding RNAs, ultimately affecting the phenotype (Baig et al., 2016). Mutations in SNPs are caused by environmental factors and spontaneous mutations in DNA or RNA in nature (Kato et al., 2001). Gene expression levels are affected by many factors, such as SNP density, TE density, and methylation levels (Hollister and Gaut 2009). An enhanced methylation state of TEs might suppress the expression of neighboring genes in autotetraploid rice, suggesting that chromosome doubling induces methylation variation in TEs that affect gene expression (Zhang et al., 2015a). The methylation status of TEs near genes likely plays a role in the biased expression of genes depending on the subgenome location (Hollister and Gaut 2009; Springer et al., 2016). Decreased expression of genes in a submissive subgenome in the mesopolyploid crop species *B. rapa* is associated with higher transposon coverage of DNA upstream of the transcriptional unit and increased smRNA coverage of the same region (Woodhouse et al., 2014). Genes and TEs vary among subgenomes and they are associated with gene expression in *B. rapa* (Murat et al., 2014; Cheng et al., 2016). Regulatory SNPs in genes can modify gene expression levels and affect related phenotypes (Munkhtulga et al., 2010). However, little is known about correlations between SNP variations during transcription and gene expression.

Here, we investigated the asymmetric phenomenon of SNPs transmitted from DNA to RNA in two subgenomes of *B. napus* based on genomic re-sequencing data and RNA-seq data. We assessed the relationships between nucleotide variations in genomes/transcriptomes and corresponding genes expression levels and found that they showed negative correlation. Further investigation showed that transmitted SNPs had positive correlation with gene expression level, which maybe the reason why A_n_ had a higher SNP density and more abundant gene expression than those of C_n_ in *B. napus*. Our study provides novel insights with respect to how evolvability of gene expression bias between subgenomes can be boosted by transmitted SNPs in polyploidy.

## Materials and Methods

### Data Collection and SNP Identification

We investigated variations in the genomic and transcriptional profiles of two *B. napus* lines, Zhongyou821 (ZY821) and Zhongshuang11 (ZS11), which had whole-genome re-sequencing and transcriptome data from two replicates of 11 tissues. These raw data were collected from the BIG Data Center under the BioProject accession codes PRJCA000376 and PRJCA001246 (Lu et al., 2019). To identify comprehensive transcription-level SNPs, we merged all RNA-seq datasets of the same sample for variation calling. Quality was controlled by using the Fastp software with default parameters (Chen et al., 2018).

A total of 6.37 and 7.56 Gb of genomic resequencing clean bases of ZY821 and ZS11 were aligned to the reference genome (*B. napus* cv. Darmor-bzh, a French winter variety) (Chalhoub et al., 2014) using BWA-MEM v0.7.15-r1140 with default parameters (Li and Durbin 2009). Mapped reads were sorted using Samtools (Li et al., 2009), and duplicate reads of the bam alignment file were marked using Picard tools (http://broadinstitute.github.io/picard). We then used the HaplotypeCaller module of the Genome Analysis Toolkit (GATK) to identify genetic variations in DNA with default parameters (McKenna et al., 2010). Variation calling and genotyping were performed across the *Darmorbzh* reference genome. We extracted SNPs using the SelectVariants module and filtered them using the VariantFiltration module of GATK with the following parameters: clusterSize, 3; clusterWindowSize, 10; filterExpression, QUAL <30.0 || MQ < 50.0 || QD < 2.

Transcriptional variations were identified by using 111.19 and 107.85 Gb RNA-seq clean bases of ZY821 and ZS11, and variants were called in RNA-seq data using a modified pipeline (https://github.com/gatk-workflows/gatk3-4-rnaseq-germline-snps-indels). We initially used STAR (Dobin et al., 2013) to construct index files of the reference genome and mapped clean RNA-seq reads to the reference genome with default parameters. Picard tools were then used to add ReadGroup and sort the mapped reads with “AddOrReplaceReadGroups SO = coordinate RGLB = mRNA.” Then, duplicate reads were marked using the MarkDuplicates module with the parameter settings with “CREATE_INDEX = true VALIDATION_STRINGENCY = SILENT.” We eliminated Ns using the SplitNCigarReads module in GATK to maintain grouping information with the parameters of rf as ReassignOneMappingQuality, RMQF 255, RMQT 60, and -U ALLOW_N_CIGAR_READS. We then identified transcriptional variations using the parameters of “-dontUseSoftClippedBases-stand_call_conf 20” in HaplotypeCaller. Finally, transcription-level SNPs were extracted and filtered by GATK using the same module and parameters as the genomic SNPs. All SNP datasets were annotated using SnpEff v4.1 (Cingolani et al., 2014) with upstream and downstream interval lengths set to 0.

### Statistical Analyses of Asymmetrical SNP Transmission During DNA Transcription

To investigate the differences between the two subgenomes of *B. napus*, we compared SNP transmission between ZY821 and ZS11. Two samples or two levels containing a common homozygous SNP were regarded as having been transmitted. We were initially concerned with the transmission of genomic SNPs at the transcriptional level in ZY821 and ZS11. Because ZY821 is one of parents of ZS11, some ZY821 genetic components were transferred to the ZS11 genome during breeding. We further investigated the subgenome differences in SNPs transmitted from the ancestral parent to the offspring. We analyzed two types of transmission from ZY821 to ZS11, they were genomic SNP of ZY821 transferred to ZS11 genomic level and ZY821 genomic SNP transferred to ZS11 genomic level then transferred to ZS11 transcriptional level, respectively. The transmission ratio/rate was defined as the number of transmitted SNPs divided by the total number of genomic SNPs detected in the gene. In order to study the asymmetric distribution of transmitted SNPs in two subgenomes of *B. napus*, we analyzed the transmitted SNPs of all syntenic genes between A_n_ and C_n_ subgenomes (Chalhoub et al., 2014). Ratios of transmitted SNPs on A_n_ and C_n_ in different samples/levels were compared using two-tailed t-tests. A homozygous variant that arises from DNA to RNA transmission is regarded as a novel SNP variation in DNA to RNA.

### Gene Ontology Enrichment Analyses

Pathways and biological functions of genes without SNPs and genes with genomic SNPs that were all transmitted to the transcriptional level were explored via GO enrichment analyses using the PlantRegMap database (http://plantregmap.gao-lab.org/go.php) with the parameter settings of species, *B. napus*; aspects, biological process, molecular function, cellular component; threshold, and q ≤ 0.01. The GO enrichment results are shown on the ImageGP website (http://www.ehbio.com/ImageGP/).

### Gene Expression and Linear Regression Analyses

Filtered high-quality clean RNA-seq data from different tissues and periods of ZY821 and ZS11 were mapped to the “*Darmor-bzh*” genome (Chalhoub et al., 2014) using STAR with default parameters (Dobin et al., 2013). The mapped reads with mapping quality (MQ) ≤ 30 were filtered using Samtools, and bam files were sorted. As TPM (transcripts per kilobase of exon model per million mapped reads) could correct the inconsistences while comparing the RNA-seq abundance among independent samples (Wagner et al., 2012), StringTie (Pertea et al., 2015) was used to count unique and normalized mapped reads as TPM for each gene with the parameter of “-B -e -G”.

We used linear regression analyses to investigate correlations between gene expression level and SNP numbers in gene regions, new SNP variations in DNA to RNA, and SNP transmission rates, respectively. Genes with different transmission rates were divided into 10 groups at 0.1 intervals. Genes with different numbers of SNP variations were divided into groups of 10 intervals in ZY821 DNA, ZY821 RNA, and ZS11 RNA, whereas those in ZS11 RNA were arranged into groups with 20 intervals since SNP variations occurred frequently. The linear model is y_i_ = ax_i_+b, where y_i_ is the average TPM of the *i*th group, and x_i_ is a different group.

## Results

### Identification and Annotation of SNPs in ZY821 and ZS11

We filtered 6.37 Gb of genome re-sequencing, and 111.19 Gb RNA-seq data were used to identify SNP variation in ZY821. In total, 1,178,526 SNPs were detected in ZY821 genomic DNA, along with 19 chromosomes and 21 scaffolds (Supplementary Table S1). We found that 83,179 SNPs in chromosome chrC03 had the most mutations. Among these SNPs, there were 349,509 SNPs distributed in 40,903 genic regions and 846,691 SNPs located in intergenic regions. 162,124 of the genic SNPs were located in exon regions and 139,244 SNPs in intron regions (Supplementary Table S2). Among the SNPs in exon regions, 69,526 missense and 91,469 synonymous variations were annotated. We also identified 792,189 SNPs at the transcription level in ZY821, and most variations were detected in chromosome chrA03 (63,925 SNPs). We found 536,701 and 144,456 variations in the exon and intron regions, respectively, and 64.05% (349,110) and 34.63% (185,846) of SNPs in exon regions were annotated as synonymous and missense variants, respectively (Supplementary Table S2).

After quality control, we identified 1,823,207 and 686,858 genomic and transcriptional SNPs, respectively, in ZS11 using 7.56 Gb genome re-sequencing and 107.85 Gb RNA-seq data. Among the genomic SNPs, 1,352,662 were located in intergenic regions with the most variations, and 228,122 and 199,500 were located in exons and introns, respectively. Overall, 97,806 (42.87%) and 128,632 (56.39%) SNPs in exons were annotated as missense and synonymous variants, respectively. Among the SNPs at the transcription level, 464,923 and 126,513 variations occurred in the exon and intron regions, respectively. Among the SNPs in exons, 163,198 (35.10%) and 300,178 (64.57%) were missense and synonymous variants, respectively (Supplementary Table S2).

We identified 27,024 shared genes without SNPs at the genomic and transcriptional levels in both ZY821 and ZS11, among which 8,756 and 18,268 were located on A_n_ and C_n_, respectively. Results of GO enrichment analysis of the genes without SNPs showed that 54 terms were enriched in cellular components, biological processes, and molecular functions. Almost all terms were basic biological processes for growth, such as an integral component of membrane and membrane part in cellular component, the regulation of nucleic acid-templated transcription and nucleoside bisphosphate metabolic processes in biological processes, enzyme inhibitor activity, and electron carrier activity in molecular function (Supplementary Table S3), which indicated that these genes were conserved.

### Asymmetric Distribution of SNPs Between A_n_- and C_n_-Subgenomes

**FIGURE 1:**
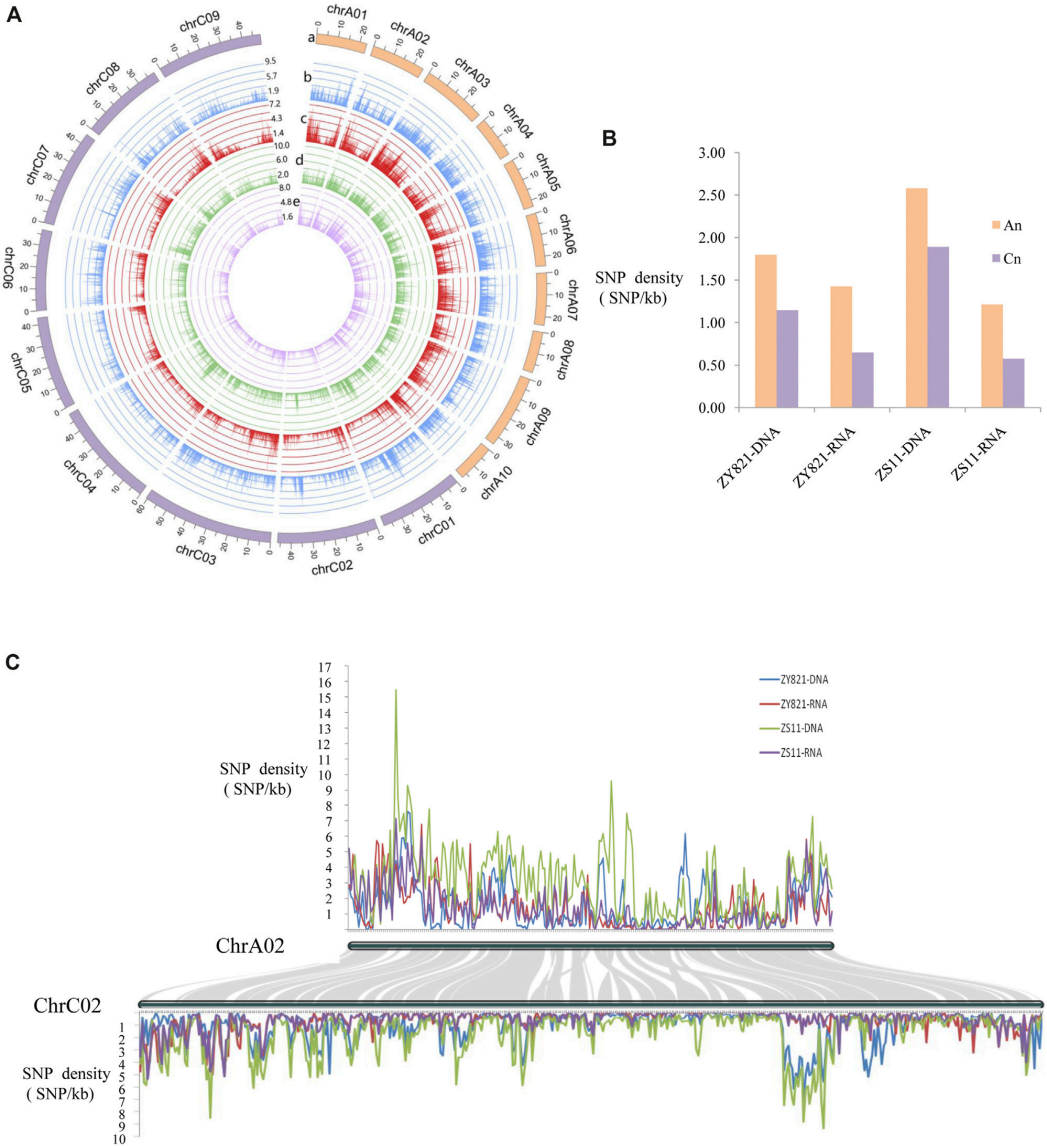
Distribution of SNP density on DNA/RNA levels of two *B. napus* lines ZY821 and ZS11 **(A)** Density of SNPs in 19 chromosomes among four levels. a: Sizes (Mb) of 19 chromosomes in *B. napus*; b–e: line graph of SNP density (y-axis, numbers of SNPs/kb) for chromosomes at ZY821 and ZS11 genomic and transcriptional levels, respectively. **(B)** Distribution of SNP density in two subgenomes. **(C)** Local comparison of SNP density in chromosomes chrA02 and chrC02. Connecting gray lines in background highlight conserved syntenic genes between chrA02 and chrC02.

The asymmetric characteristics of the A_n_ and C_n_ subgenomes of *B. napus* have been identified (Qian et al., 2014; Lu et al., 2019; Wu et al., 2019). We found that ZY821 and ZS11 had more total SNPs in C_n_ than in A_n_ at the genomic level, whereas C_n_ had fewer SNPs than A_n_ at the transcriptional level (Supplementary Table S1). The total length of A_n_ was 315.05 Mb in the reference genome, which was less than that in the C_n_ (526.93 Mb) subgenome; therefore, we assessed SNP density to reevaluate differences between the two subgenomes. Figure 1A shows the distribution of SNP densities in the whole genome. The density of ZS11 genomic SNPs was the highest at 3.7/kb in scaffold A10_random and that of ZS11 transcriptional SNPs was the lowest (0.18/kb) in the scaffold Unn_random. These might have been due to the frequency of coding genes being the lowest (1/15.61 kb) in the scaffold Unn_random. The mean SNP density in the genome was 1.39 and 0.93/kb at the ZY821 genomic and transcriptional levels, and 2.14, and 0.81/kb at the ZS11 genomic and transcriptional levels (Supplementary Table S1). The genomic and transcriptional SNP density was higher in A_n_ than in C_n_, especially at the transcriptional level, and the SNP density of A_n_ was almost twice that of C_n_ (Figure 1B). Taken chromosomes chrA02 and chrC02 as an example due to they had good collinearity, the SNP density of chrA02 was generally higher than that of chrC02, although that of chrA02 was occasionally lower than that of chrC02 in some chromosomal regions (Figure 1C).

### Asymmetric SNP Transmission Between A_n_- and C_n_-Subgenomes

**FIGURE 2:**
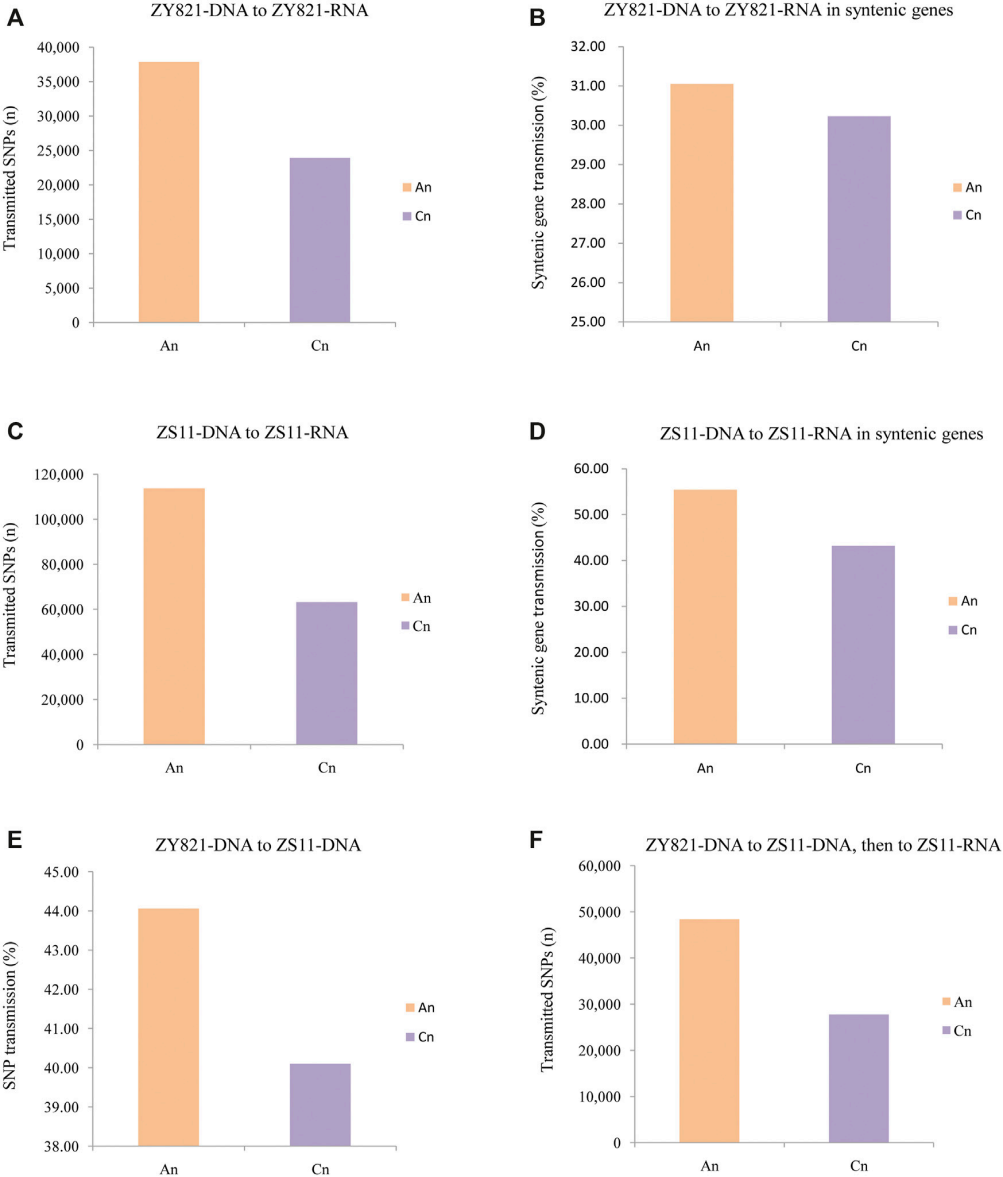
Statistical analyses of transmitted SNPs in two subgenomes at different levels in two *B. napus* lines ZY821 and ZS11. **(A)** Rates of ZY821 genomic SNPs transmitted to the ZS11 genome. **(B)** Numbers ofZY821 genomic SNPs transmitted to theZY821 transcriptional level. **(C)** Rates of SNPsin syntenic ZY821 genes transmitted from genome to theZY821 transcriptional level. **(D)** Numbers of SNPs transmitted from the genomic to the transcriptional level in ZS11. **(E)** Rates of genomic SNPs in syntenic genes of ZS11 transmitted to the transcriptional level. **(F)** Numbers of genomic SNPs from ZY821 transmitted to the ZS11 genome and conserved at the transcriptional level in ZS11.

We initially detected 61,832 genomic SNPs that were transmitted at the transcriptional level in ZY821 and involved 25,583 coding genes. A total of 37,845 and 23,920 SNPs occurred in A_n_ and C_n_, respectively (Figure 2A), whereas 14,258 synonymous SNPs in A_n_ while 6,952 synonymous SNPs in C_n_. Among syntenic genes in A_n_ and C_n_, the SNP transmission ratio from DNA to RNA was 31.05% in A_n_ and 30.23% in C_n_ (Figure 2B). In ZS11, 113,752 SNPs were transmitted from DNA to RNA in A_n_, and 63,245 in C_n_ included 35,774 coding genes (Figure 2C). The ratios of transmitted syntenic genes in A_n_ and C_n_ were 55.43 vs. 43.21% (*p* = 5.45 × 10^−89^; Figure 2D). Parental-related transmitted SNPs differed between the two subgenomes. Among 492,916 genomic SNPs from ZY821 transmitted to ZS11, in total 249,185, 241,871, and 1,860 were in A_n_, C_n_, and the scaffold Unn_random, respectively. These findings indicated that 44.06% of genomic SNP in ZY821was transmitted to the A_n_ of ZS11, and 40.10% of SNPs in C_n_ of ZY821 were transmitted to C_n_ of ZS11 (Figure 2E). Moreover, 76,188 SNPs detected at the ZY821 genomic level were transmitted to the ZS11 genome, followed by the ZS11 transcriptional level; of these, 48,367 (63.48%) of 76,188 occurred in A_n_ and 27,751 (36.42%) in C_n_, respectively (Figure 2F). These results indicated that SNPs in A_n_ were more prone to transmission than those in C_n_, especially transmission SNPs from the genomic level transfer to the transcriptional level.

**FIGURE 3:**
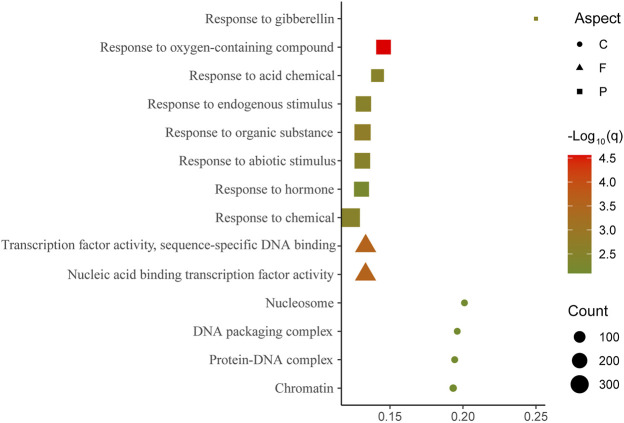
Gene ontology (GO) enrichment findings of genes with 100% of transmitted SNPs from genomic to transcriptional level in ZS11 False discovery rate (q) of all the enriched GO terms was <0.01. • cellular component (C); ▴ molecular function(F); ■ biological process (P). Larger symbols indicate increased gene expression levels.

The results of GO enrichment analyses revealed the functions of genes with a high ratio of SNP transmission. All (100%) genomic SNPs in 3,967 genes were transmitted to the transcriptional level in ZY821. The number of transmitted genes did not substantially differ between A_n_ and C_n_ (1,987 vs. 1,971). Only three GO terms were enriched in the biological process, viz. response to endogenous stimulus, response to hormone, and response to organic substances (Supplementary Table S4). Excluding biological processes, no GO terms were enriched in molecular function and cellular components (q < 0.01). Among 9,018 completely transmitted genes detected in ZS11, 5,171 and 3,835 were located in A_n_ and C_n_, respectively. Eight GO terms were enriched in biological processes, four in cellular components, and two in molecular functions (Figure 3; Supplementary Table S5), which are involved in fundamental biological processes and stress responses. All three GO-enriched terms in the biological process of ZY821 were also enriched in ZS11, indicating that these genes play essential roles in the speciation of ZS11.

### Impact of Transmitted SNPs on Asymmetrical Gene Expression

**FIGURE 4:**
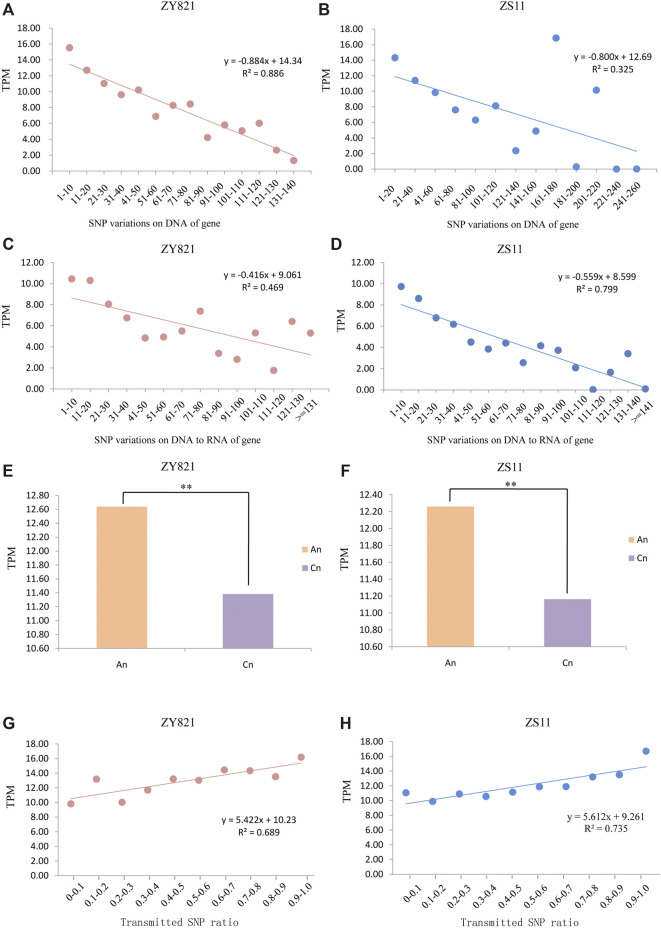
Correlations of the gene expression levels with SNP variations and transmission ratio Correlations and linear regression between gene expression (TPM) and numbers of genomic SNP in all ZY821 **(A)** and ZS11 **(B)** genes. Correlations and linear regression analyzes between TPM and numbers of novel SNP variations on DNA to RNA of gene in ZY821 **(C)** and ZS11 **(D)**. Average expression levels of all syntenic genes in A_n_ and C_n_ in ZY821 **(E)** and ZS11 **(F)**, ** represent *p* < 0.01. Correlations and linear regression analyzes between TPM and ratios of SNPs of all genes transmitted from DNA to RNA in ZY821 **(G)** and ZS11 **(H)**.

We analyzed whole gene expression levels of ZY821 and ZS11 to determine correlations between rates of SNP transmission from DNA to RNA and gene expression. We merged RNA-seq data from different tissues and periods and found that 14,717 and 15,822 genes were not expressed in ZY821 and ZS11, respectively, and 10,931 genes of them were shared, which might be because they were tissue- and time-specific. Gene expression levels decreased as the number of genomic SNPs in genes increased (Figures 4A,B), indicating that the expression of a gene with more SNPs would be lower in the *B. napus* genome. The expression of genes containing more RNA variations was lower in ZY821 and ZS11 during transcription (Figures 4C,D), which was consistent with the number of variations at the genomic and gene expression levels. However, SNP Figures 4E,F density was higher in A_n_ (Figure 1B), but gene expression was higher in C_n_ (Figures 4E,F). Further results revealed that transmitted SNPs had significantly associated with gene expression level. We found that 100% of 3,967 and 9,018 genes with SNPs from the genomic level were transmitted to the transcriptional level in ZY821 and ZS11, respectively. We established a linear regression model to fit the details of the SNP transmission ratio from DNA to RNA and gene expression. SNP transmission rates were significantly and positively associated with the gene expression levels. The *R* ^2^ values of the fitted line were 0.689 and 0.735 in ZY821 and ZS11, respectively (Figures 4G,H), indicating that gene expression increased as the SNP transmission ratio from the DNA to the RNA level increased. These results showed that the ratio of SNPs transmitted from DNA to RNA correlated well with gene expression. The rates and numbers of transmitted SNPs were higher in A_n_ than in C_n_ (Figure 2), signifying asymmetric gene expression in polyploid *B. napus*.

## Discussion

Polyploidization is considered the primary driver of plant species diversification and plays an important role in plant genome evolution, adaptive selection, gene function innovation, and crop domestication (Soltis et al., 2016). Polyploidy is a state in which the cells of an organism contain two or more sets of ancestral genomes, which may not be accompanied by multiple complete sets. Therefore, redundant genes or components in polyploidy might mutate or recombine into different neomorphic mutants with a reduced risk of extinction. Fractionation, subfunctionalization, and neofunctionalization are significant outcomes for redundant genes. Subgenome dominance develops when a subgenome expresses more genes than another when coexistent within cells (Cheng et al., 2016). The rapid development of genome sequencing and analysis technologies has led to more sequenced genomes and identified polyploidization events that have facilitated comparative genomic studies on polyploid evolution. In polyploidy *B. napus*, A_n_ contains a higher density of coding genes, transposable elements, and more diversity than C_n_ (Chalhoub et al., 2014; Wu et al., 2019). We showed a higher SNP density and more transmitted SNPs from DNA to DNA/RNA in A_n_, especially from the DNA level to the RNA level, which complements the asymmetric feature in polyploidy. The asymmetric features in the two subgenomes might be due to more frequent outcrossing between *B. napus* and *B. rapa* than between *B. napus* and *B. oleracea* in terms of breeding behaviors (Wu et al., 2019). The A_n_ subgenome of a modern *B. napus* could retain excellent variations in artificial selection according to the aims of breeders. The GO findings showed that genes with completely transmitted SNPs of ZY821 and ZS11 were commonly enriched in fundamental biological processes and stress responses. This might be an excellent stress SNP micromutation in coding genes that facilitate disease resistance. Therefore, they have been selected by breeders considering adaptability and disease resistance and they were widely planted in China during recent decades.

Gene expression is the most fundamental level at which a genotype produces observable traits. The regulation of gene expression is the basis for cellular differentiation, development, morphogenesis, versatility, and adaptability of all organisms. The amount of gene expression level was affected by SNP density, TE density, and methylation level. Regulatory factors containing a splice acceptor and promoter in intergenic regions are important for regulating gene expression (LeBowitz et al., 1993; Mette et al., 2000; Weber et al., 2007). Different profiles of methylation and altered splicing forms are also associated with gene expression levels (Liu et al., 2014; Mei et al., 2017; Renny-Byfield et al., 2017). Transposons can be repressed, and the expression of genes encoding proteins can be affected by RNA-directed DNA methylation (Rowley et al., 2017). The present findings showed that the numbers and transmission rates of SNPs were associated with gene expression. Changes in SNP bases might result in different combinations of transcription factors, which would lead to different levels of coding gene expression. Transposable elements, splice forms, methylation modifications, and SNP density contribute to gene expression, but systematic understanding remains limited. The integration of genomics, transcriptomics, epigenetics, morphology, and synthetic methods in appropriate species is essential for gaining further insight.

Transcription factors are proteins that control the rates at which genetic information is transcribed from DNA to messenger RNA by binding to a specific DNA sequence, and they are essential for regulating gene expression. Mutations in transcription factors may affect the expression of related genes. A specific binding SNP in the first intron of the *SPP1* gene affects the functional characteristics of *SPP1* at the DNA and RNA levels by activating abnormal splicing of the first intron (Muráni et al., 2009). A changed base in rs3122605-G upregulated *IL10* gene expression and increased the risk of systemic lupus erythematosus in European Americans (Sakurai et al., 2013). Our findings showed that gene expression decreased with increasing DNA (or DNA to RNA) variations in coding genes. This indicated that more variations at the level of DNA and during transcription from DNA to RNA resulted in lower gene expression. We excluded high-density variations that occur due to fewer mismatch mapping transcriptional reads, and determined the SNP frequency within 150 bp windows (the read length of RNA-seq in this study) in the entire genome (5,668,614 windows). We found that only 68 and 38 windows in ZY821 and ZS11 contained more than 10 SNPs (default mismatch mapping parameter of STAR), indicating that genes with a higher density of RNA variation did not occur due to a few mismatch reads. This may be because more genomic SNP variations in the gene would increase the risk of a transcription factor not binding to a specific DNA sequence, and nucleotide variations during the transcription process from DNA to RNA might affect RNA alternative splicing and transcriptional inhibition. Alternative splicing of precursor mRNAs from multiexon genes allows an organism to increase its coding potential and regulate gene expression through multiple mechanisms (Reddy et al., 2013). We found 19,756 and 17,509 RNA SNPs in alternative splice regions in ZY821 and ZS11, respectively. These SNPs may affect gene expression via alternative splicing.

Subgenome expression dominance might be obscured by potential homoeologous exchange between different subgenomes, for example, in wheat (Pfeifer et al., 2014; Harper et al., 2016) and *B. napus* (Chalhoub et al., 2014). We found lower expression of genes with more SNP variations, but A_n_ had not only a higher SNP density, but also a higher level of gene expression. Further analyses revealed that the transmitted SNP ratio was positively correlated with gene expression level. The SNPs transmitted from DNA to RNA might be excellent variations that facilitated the formation of traits that were favored and selected by breeders, and finally retained. Genome dominance is heritable; the expression of a gene in a dominant subgenome tends to be higher than that of its recessive homeolog (Woodhouse et al., 2014). Transmitted SNPs vary in homologous genes in the corresponding subgenome and might be associated with gene dominance. Given our understanding of the subgenome dominance of *B. napus*, the selected SNPs transmitted from DNA to RNA played important roles in gene expression, but the random SNPs involved in down-regulating gene expression might obscure gene expression dominance. In this study, we used the statistical methods to discover a positive correlation between transmitted SNPs and gene expression level, besides this, the effect of transmitted SNPs on gene expression level and subgenome dominance needs to be verified in more polyploidy species and direct molecular experiment evidence. Asymmetric divergence and genome evolution are popular topics in plant science, especially in polyploidy. The innovative knowledge of transmitted SNPs might be an important field in genome evolution of polyploid crops.

## Conclusion

We identified variations in genomic and transcriptional SNPs in ZY821 and ZS11 and investigated the correlation between SNP numbers/transmission ratios and gene expression. We showed that variations in DNA or the transcription process from DNA to RNA correlated negatively with gene expression levels. The extent of gene expression increased with an increasing ratio of SNP transmission from DNA to RNA, which might explain why A_n_ had a higher SNP density and more abundant gene expression. SNPs transmitted from DNA to DNA or DNA to RNA might play important roles in asymmetrical evolution and subgenome dominance in polyploidy.

## Data Availability

The original contributions presented in the study are included in the article/Supplementary Material, further inquiries can be directed to the corresponding authors.
